# Supplementary material for: Genomic Surveillance of COVID-19 Variants With Language Models and Machine Learning
Source: Front Genet. 2022 Apr 8;13:858252. doi: 10.3389/fgene.2022.858252 (PMC9024110; doi:10.3389/fgene.2022.858252)
Supplement: Supplementary file 1 [file DataSheet1.pdf]

Supplementary Information

Supplementary text

Entropy as a measure of the information content of the latent dimensions

We hypothesized that mutations increase the chaotic dynamics in the latent space of spike genes. To calculate entropy, we used the accelerated versions of the Approximate Entropy and Sample Entropy algorithms, called Fast Approximate Entropy and Fast Sample Entropy (Tomčala, 2020). Both algorithms aim to quantify how often different patterns of data are found in a time series. Fast Approximate Entropy, however, is a biased statistic and depends on the length of the series. Since we could have different counts of genome sequences collected each month, we preferred Sample Entropy, which is independent of the length of the series.

Monthly entropy and DCCA to model the new cases

Detrended Cross-Correlation Analysis (DCCA) was performed between the entropy dimensions and the new cases (Prass & Pumi, 2020b). DCCA is a modification of the standard cross-correlation analysis for finding relationships between non-stationary time series. High cross-correlation for different lead periods revealed that the entropy values in a given month could be used to predict the new cases in different countries in subsequent months. Different countries had different lead times at which the highest cross-correlation was observed between the entropy dimensions and the cases, ranging from 1-6 months. Overall, a lead time of two months was chosen to model the new cases.

A similar analysis was done with daily values of entropy and new cases. Entropy was calculated in rolling windows, and cross-correlation analysis was performed between entropy and new cases at different lead periods. Although the cross-correlation values were found to be significant, the values were low and ranged between -0·1 to 0·1. Therefore, we decided to use the monthly entropy values for the modeling exercise.

Prediction of new cases with 'blips'

We also experimented with "blips" as a feature to predict the new cases. Blips are sudden changes in the values of the latent dimensions. These changes may be caused by a mutation, which changes the words (codons) in a given genome sequence. This hypothesis was validated in simulation experiments in synthetic datasets. Each dimension of the spike gene embeddings for a country was analyzed for the presence of temporal anomalies. Countries having a minimum of 20 samples in any given month were selected and the same number of records (minimum samples in any given month for that country) were sampled without replacement from each month. These records were used to define control limits of ±1 standard deviation from the mean value for each dimension, and all values in the full dataset

outside those limits were categorized as 'Blip' points. Blip counts in each month were normalized by calculating the number of blips per sample collected in a month for each dimension (normalized blips). The embedding dimensions were then compared in terms of the total normalized blips for each country to observe the significant dimensions and dis(similarity) in trends among different countries. Cumulative counts of normalized blips were analyzed to understand the temporal accumulation of blips in each dimension. Similar to entropy, blips were found to have a leading relationship with the cases. However, regression modelling results with sample entropy were found to be better.

Strainflow Dashboard

Implementation: The strainflow dashboard web application is primarily built using *ReactJS* and other accompanying libraries for UI needs and *GraphJS* for graphical needs. Python libraries such as numpy, pandas, matplotlib and seaborn were used to pre-process and infer the dataset. The Random Forest regression model was implemented using the R library *randomForest*. The web application is available for use on http://strainflow.tavlab.iiitd.edu.in and works on all modern browsers.

Functionalities: The application has three tabs: Cases Plots, Entropy Plots, and Paper. The Cases Plot tab exhibits two graphs; one compares the actual number of cases with our predicted cases, with a two-month lead time, while the second shows entropy against the caseload for a given country. The Entropy Plots tab displays the sum of sample entropy across all the latent dimensions for each pair of countries. The toggler present above the graph can be used to change countries to compare their entropies. Lastly, the "Paper" tab presents a graphical abstraction of our paper.

Discussion: COVID-19 had a devastating impact on our health systems, thus with caseload predictions made two months in advance, we provide a data-driven handle on epidemiological surveillance to warn about potential upcoming case surges, so that people can be prepared in advance and appropriate preemptive steps can be taken by policymakers to prevent the spread of COVID-19.

Supplementary Figures

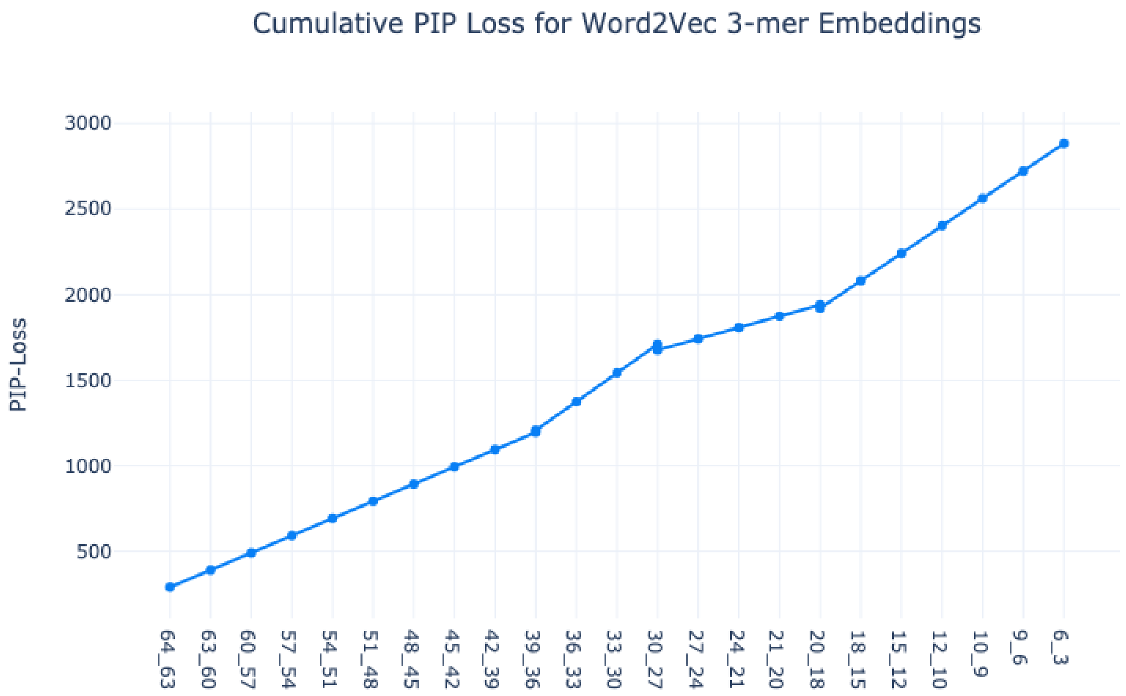

Figure S1. ***Determination of optimal dimensions in Word2Vec model****: Cumulative PIP Loss computed between word2vec codon embeddings differed by vector size taken in multiples of 3, till dimension size 64.*

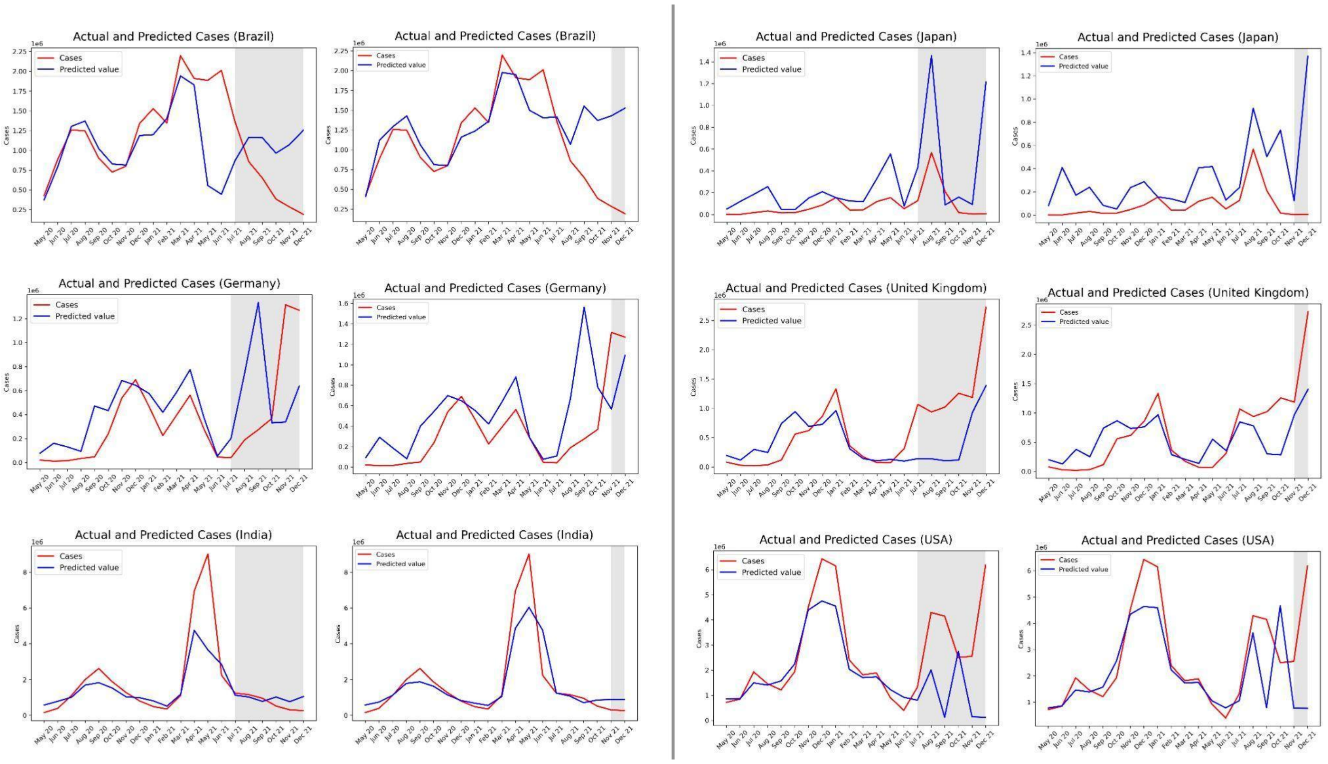

Figure S2. ***Comparison of regression model frozen in June 2021 compared to the one frozen in October 2021 for all countries.***

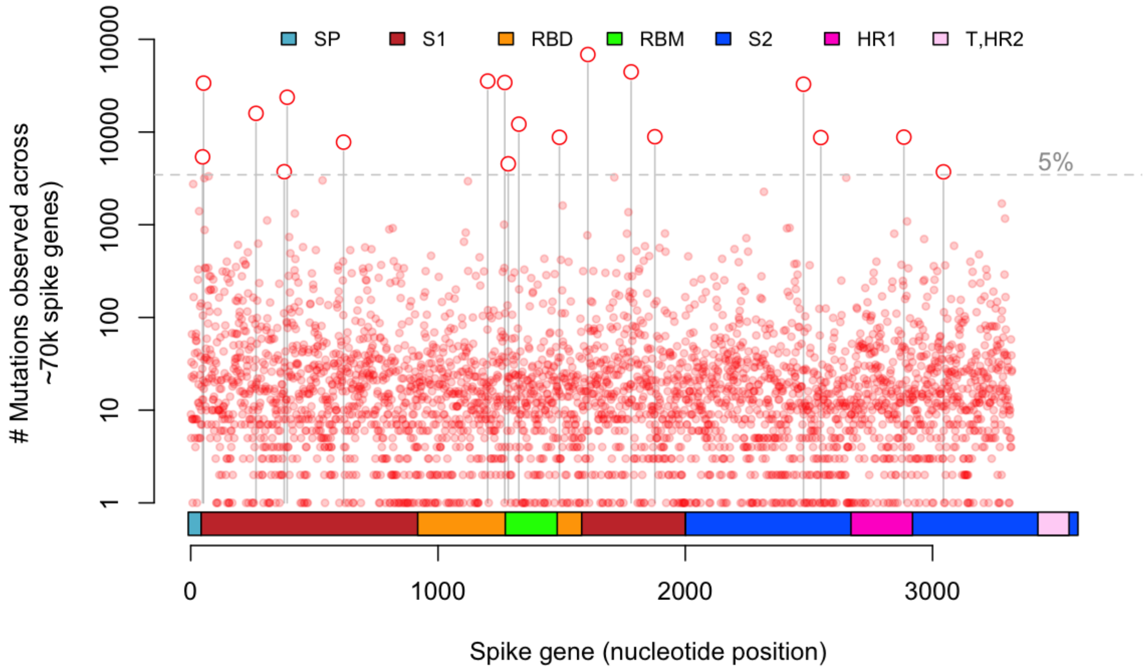

Figure S3. ***Spike gene domain structure and associated mutations till Jan, 2022.***

Supplementary Tables

Country	Pearson Correlation	p value	Spearman's Correlation	p value
USA	0.93	2.81 x 10^{-6}	0.94	0.00
England	0.82	5.60 x 10^{-4}	0.71	8.14 x 10^{-3}
France	0.8	9.36 x 10^{-4}	0.55	5.25 x 10^{-2}
Germany	0.76	2.47 x 10^{-3}	0.77	2.92 x 10^{-3}
India	0.71	7.05 x 10^{-3}	0.59	3.60 x 10^{-2}
Japan	0.68	1.08 x 10^{-2}	0.57	4.73 x 10^{-2}
Brazil	0.67	1.22 x 10^{-2}	0.78	2.62 x 10^{-3}

Table S1. ***Pearson and Spearman Correlation coefficients between the delta of predicted and actual cases in different countries.***

Latent Dimensions from Word Embeddings

32		28		13		3		15		30	
**3-mers**	**Weights**	**3-mers**	**Weights**	**3-mers**	**Weights**	**3-mers**	**Weights**	**3-mers**	**Weights**	**3-mers**	**Weights**
TAA	0.64	CTG	0.93	ACG	0.9	TAA	1.02	ACG	0.84	TAG	1.11
CCC	0.49	TCG	0.93	TCG	0.7	GGG	0.83	CGG	0.81	GGG	0.62
GCG	0.49	CCC	0.92	AGC	0.65	AGC	0.77	CCC	0.71	CAT	0.55
GGC	0.48	CAC	0.91	TAA	0.61	CGC	0.75	CGC	0.5	TAA	0.53
CTG	0.48	TAA	0.74	GCG	0.53	ACG	0.69	CGT	0.42	CTG	0.5
AGA	0.4	TAC	0.65	TCC	0.51	TGA	0.58	AGG	0.35	TCG	0.46
CCG	0.37	AGC	0.6	AGG	0.47	TAG	0.53	TAG	0.33	CAC	0.45
CGG	0.34	TTC	0.55	TGC	0.35	TCG	0.53	GGA	0.3	ACC	0.35
CTA	0.33	CGC	0.55	AGT	0.32	CGG	0.52	CTC	0.29	ACG	0.33
GTG	0.3	AGA	0.5	CGG	0.32	CAC	0.42	GGC	0.28	CTA	0.3

Table S2: Dimensions of Concern and top 10 codons with their absolute weights.

DOC	Rank	Codon	Amino Acid	Variant
32	5	CTG	(Leu/L) Leucine	L452R
	6	AGA	(Arg/R) Arginine	T19R, R158G
	8	CGG	(Arg/R) Arginine	L452R
27	1	AGC	(Ser/S) Serine	R190S
	7	CTG	(Leu/L) Leucine	L452R
	10	CGG	(Arg/R) Arginine	L452R
28	1	CTG	(Leu/L) Leucine	L452R

	4	CAC	(His/H) Histidine	Q1071H, D1118H, Q677H
	7	AGC	(Ser/S) Serine	R190S
	8	TTC	(Phe/F) Phenylalanine	V367F, Del157
	10	AGA	(Arg/R) Arginine	T19R, R158G
17	1	CGG	(Arg/R) Arginine	L452R
	2	CTG	(Leu/L) Leucine	L452R
	6	ACG	(Thr/T) Threonine	K417T
	10	GCC	(Ala/A) Alanine	A475H
2	2	CGG	(Arg/R) Arginine	L452R
	3	CTG	(Leu/L) Leucine	L452R
	9	CAC	(His/H) Histidine	Q1071H, D1118H, Q677H
22	3	ACG	(Thr/T) Threonine	K417T
	8	AGT	(Ser/S) Serine	R190S
	9	CGG	(Arg/R) Arginine	L452R
13	1	ACG	(Thr/T) Threonine	K417T
	7	AGG	(Arg/R) Arginine	R190S
	9	AGT	(Ser/S) Serine	R190S
	10	CGG	(Arg/R) Arginine	L452R
25	3	CTG	(Leu/L) Leucine	L452R
	7	AGA	(Arg/R) Arginine	T19R, R158G
	8	GCC	(Ala/A) Alanine	A475H
	10	AGG	(Arg/R) Arginine	R190S
5	1	CTG	(Leu/L) Leucine	L452R
	4	CGT	(Arg/R) Arginine	P681R
	5	CAC	(His/H) Histidine	Q1071H, D1118H, Q677H
	6	GCC	(Ala/A) Alanine	A475H
	9	AGC	(Ser/S) Serine	R190S
	10	TTC	(Phe/F) Phenylalanine	V367F, Del157
7	4	GCC	(Ala/A) Alanine	A475H
	6	ACG	(Thr/T) Threonine	K417T
	7	CGT	(Arg/R) Arginine	P681R
	10	CAG	(Gln/Q) Glutamine	Q677H
3	3	AGC	(Ser/S) Serine	R190S

	5	ACG	(Thr/T) Threonine	K417T
	9	CGG	(Arg/R) Arginine	L452R
	10	CAC	(His/H) Histidine	Q1071H, D1118H, Q677H
15	1	ACG	(Thr/T) Threonine	K417T
	2	CGG	(Arg/R) Arginine	L452R
	5	CGT	(Arg/R) Arginine	P681R
	6	AGG	(Arg/R) Arginine	R190S
30	3	CAT	(His/H) Histidine	H655Y, P681H, Q677H, Del 69, Q1071H
	5	CTG	(Leu/L) Leucine	L452R
	7	CAC	(His/H) Histidine	Q1071H, D1118H, Q677H
	8	ACC	(Thr/T) Threonine	T20N
	9	ACG	(Thr/T) Threonine	K417T

Table S3. ***Dimensions of Concern (DOCs), associated codons, and variants.***

Variants	Genomic Substitution	Reference codon	Replaced codon
L452R	T22917G	CTG	CGG
K417T	A22812C	AAG	ACG
R190S	G22132T	AGG	AGT
Q1071H	A24775T	CAA	CAT
D1118H	G24914C	GAC	CAC
Q677H	G23593T	CAG	CAT
W152C	G22018T	TGG	TGT
T716I	C23709T	ACA	ATA
D614G	A23403G	GAT	GGT
H655Y	C23525T	CAT	TAT
P681H	C23604A	CCT	CAT
A570D	C23271A	GCT	GAT
G142D	G21987A	GGT	GAT
D138Y	G21974T	GAT	TAT

S982A	T24506G	TCA	GCA
V1176F	G25088T	GTT	TTT
T478K	C22995A	ACA	AAA
K417N	G22813T	AAG	AAT
T20N	C21621A	ACC	AAC
T19R	C21618G	ACA	AGA
E484Q	G23012C	GAA	CAA
E484K	G23012A	GAA	AAA

Table S4. ***List of variants, corresponding genomic substitution, and associated codon change.***

RESOURCE	SOURCE	IDENTIFIER
Data		
SARS-CoV-2 genome sequeces	GISAID	https://www.gisaid.org/
COVID-19 cases	GitHub	https://github.com/CSSEGISandData/COVID-19
Software and algorithms		
CoV-Seq	GitHub	https://github.com/boxiangliu/covseq
Flt-SNE (version 1.2.1)	GitHub	https://github.com/KlugerLab/FIt-SNE
Anaconda (version 4.10.1)	anaconda.com	https://www.anaconda.com/
Python (version 3.8.5)	python.org	https://www.python.org/downloads/release/python-385/
biopython (version 1.78)	PyPI	https://pypi.org/project/biopython/1.78/
gensim (version 4.0.1)	PyPI	https://pypi.org/project/gensim/4.0.1/
numpy (version 1.19.2)	PyPI	https://pypi.org/project/numpy/1.19.2/
pandas (version 1.1.3)	PyPI	https://pypi.org/project/pandas/1.1.3/
matplotlib (version 3.3.2)	PyPI	https://pypi.org/project/matplotlib/3.3.2/
seaborn (version 0.11.0)	PyPI	https://pypi.org/project/seaborn/0.11.0/
plotly (version 4.14.3)	PyPI	https://pypi.org/project/plotly/4.14.3/
R version (4.1.0)	CRAN	https://cran.r-project.org/
lubridate (version 1.7.0)	CRAN	https://cran.r-project.org/web/packages/lubridate/
dplyr (version 1.0.7)	CRAN	https://cran.r-project.org/web/packages/dplyr/
zoo (version 1.8.9)	CRAN	https://cran.r-project.org/web/packages/zoo/
tseries (version 0.10.48)	CRAN	https://cran.r-project.org/web/packages/tseries/

plyr (version 1.8.6)	CRAN	https://cran.r-project.org/web/packages/plyr/
reshape2 (version 1.4.4)	CRAN	https://cran.r-project.org/web/packages/reshape2/
ggplot2 (version 3.3.5)	CRAN	https://cran.r-project.org/web/packages/ggplot2/
ggpubr (version 0.4.0)	CRAN	https://cran.r-project.org/web/packages/ggpubr/
TSEntropies (version 0.9)	CRAN	https://cran.r-project.org/web/packages/TSEntropies/
DCCA (version 0.1.1)	CRAN	https://cran.r-project.org/web/packages/DCCA/
Boruta (version 7.0.0)	CRAN	https://cran.r-project.org/web/packages/Boruta/
randomForest (version 4.6.14)	CRAN	https://cran.r-project.org/web/packages/randomForest/
ape (version 5.5.0)	CRAN	https://cran.r-project.org/web/packages/ape/
iTOL (version 6.3)	Online	https://itol.embl.de/
Manuscript Code	GitHub	https://github.com/rintukutum/strainflow_manuscript

Table S5. ***List of softwares and packages used for our study with their sources and identifiers for the reproducibility of this study.***
